# Inflammatory arthritis and eye diseases: a Mendelian randomization study

**DOI:** 10.3389/fendo.2023.1251167

**Published:** 2023-10-09

**Authors:** Xinlin Nie, Zhaoliang Liu, Dongheng Xie, Yang Sun

**Affiliations:** Department of Orthopedic Center, The First Hospital of Jilin University, Changchun, China

**Keywords:** inflammatory arthritis, rheumatoid arthritis, ankylosing spondylitis, psoriatic arthritis, eye diseases, Mendelian randomization

## Abstract

**Objectives:**

The aim of this study was to determine causal associations between inflammatory arthritis and eye diseases (disorders of sclera, cornea, iris, and ciliary body [DSCIC] and disorders of choroid and retina [DCR]).

**Methods:**

Genome-wide association studies’ summary data of rheumatoid arthritis (RA) from a large-scale meta-analysis were used to identify genetically predicted RA. UK Biobank source data predicted ankylosing spondylitis (AS), psoriatic arthritis (PsA), and juvenile idiopathic arthritis (JIA). Furthermore, data from the FinnGen Biobank were used to identify genetically predicted eye diseases. Two-sample Mendelian randomization analysis was used to assess the causal relationship between inflammatory arthritis and eye diseases in the European population. Inverse-variance weighting (IVW) was used as the primary method, while MR-Egger, weighted median, and MR-PRESSO outlier test were used to detect heterogeneity and pleiotropy.

**Results:**

Genetically determined RA was indeed observed to have a causal effect on DSCIC (odds ratio [OR] = 1.084, *p* = 2.353 × 10^−10^) and DCR (OR = 1.151, *p* = 1.584 × 10^−19^). AS was causally associated with DSCIC (OR = 1.068, *p* < 2.024 × 10^−8^). In addition, PsA was also found to have a causal association with an increased risk of 17.9% for the development of DSCIC (OR = 1.179, *p* = 0.003). On the flip side, DSCIC increased the risk of JIA (OR = 2.276, *p* = 0.003).

**Conclusion:**

Our study provided genetic evidence for the causal associations of RA, AS, and PsA with an increased risk of DSCIC, and a causal association between RA and DCR was also identified. In addition, DSCIC greatly increased the risk of JIA.

## Introduction

Inflammatory arthritis, a disease driven by the interaction of genetic susceptibility and external environmental factors (1, 2), is characterized by synovial hyperplasia and inflammation. This category primarily encompasses rheumatoid arthritis (RA), ankylosing spondylitis (AS), psoriatic arthritis (PsA), and juvenile idiopathic arthritis (JIA) (3–5). The incidence of inflammatory arthritis has exhibited a gradual increase in recent years. It has been reported that RA, AS, and PsA collectively affect approximately 1.5% of adults (6). On the other hand, JIA poses a substantial global health burden due to its challenging diagnosis and treatment. This condition manifests with multifocal joint involvement and can affect extra-articular organs (7). As an immune-related disease, inflammatory arthritis is often associated with many extra-articular manifestations of organs, among which eye involvement is very common. Ocular signs and symptoms can sometimes serve as initial indicators, particularly in AS and RA (8, 9). Reports have suggested that 33.2% of people with AS, 7% of those with PsA, and 25%–39% of RA patients might develop corresponding eye disorders, which would further lead to serious consequences such as loss of vision or even blindness (10–13). Furthermore, it is worth noting that a substantial portion of pediatric uveitis cases are linked to JIA (14–16). A strong relationship between inflammatory arthritis and eye diseases has been investigated in several observational studies, encompassing multiple sites like the uvea, retina, sclera, and cornea (10, 17–19). Specifically, a retrospective study on U.S. patients found that RA was associated with an increased risk of scleritis (17). In addition, a cohort study from Sweden also revealed a significant association between spondyloarthritis (SpA) and anterior uveitis, especially in patients with AS (18). However, a causal conclusion cannot be drawn based solely on findings in a retrospective study or findings in several cross-sectional studies with limited sample size and confounding factors. For example, patients with inflammatory arthritis when treated with hydroxychloroquine are susceptible to the side effect of retinal toxicity, which makes medication a risk factor for ocular involvement (20, 21). Consequently, it is essential to acknowledge that this particular finding can potentially magnify the link between inflammatory arthritis and eye diseases, rendering it more challenging to establish a causal relationship between the two.

The efficacy of Mendelian randomization (MR) as a dependable technique for surmounting the constraints of observational studies and evaluating causality has been demonstrated (22). The random allocation of alleles during conception effectively regulates traditional confounding factors, leading to a well-balanced distribution of such factors across various genotypes. Furthermore, MR eliminates the potential for reverse causation, as it is biologically implausible for a disease to modify an individual’s genotype (22).

Based on statistics from publicly available genome-wide association studies (GWAS), we performed a two-sample MR analysis to investigate the causal relationship between inflammatory arthritis (i.e., RA, AS, PsA, and JIA) and eye diseases such as disorders of sclera, cornea, iris and ciliary body (DSCIC) and disorders of choroid and retina (DCR).

## Methods

**Figure 1:**
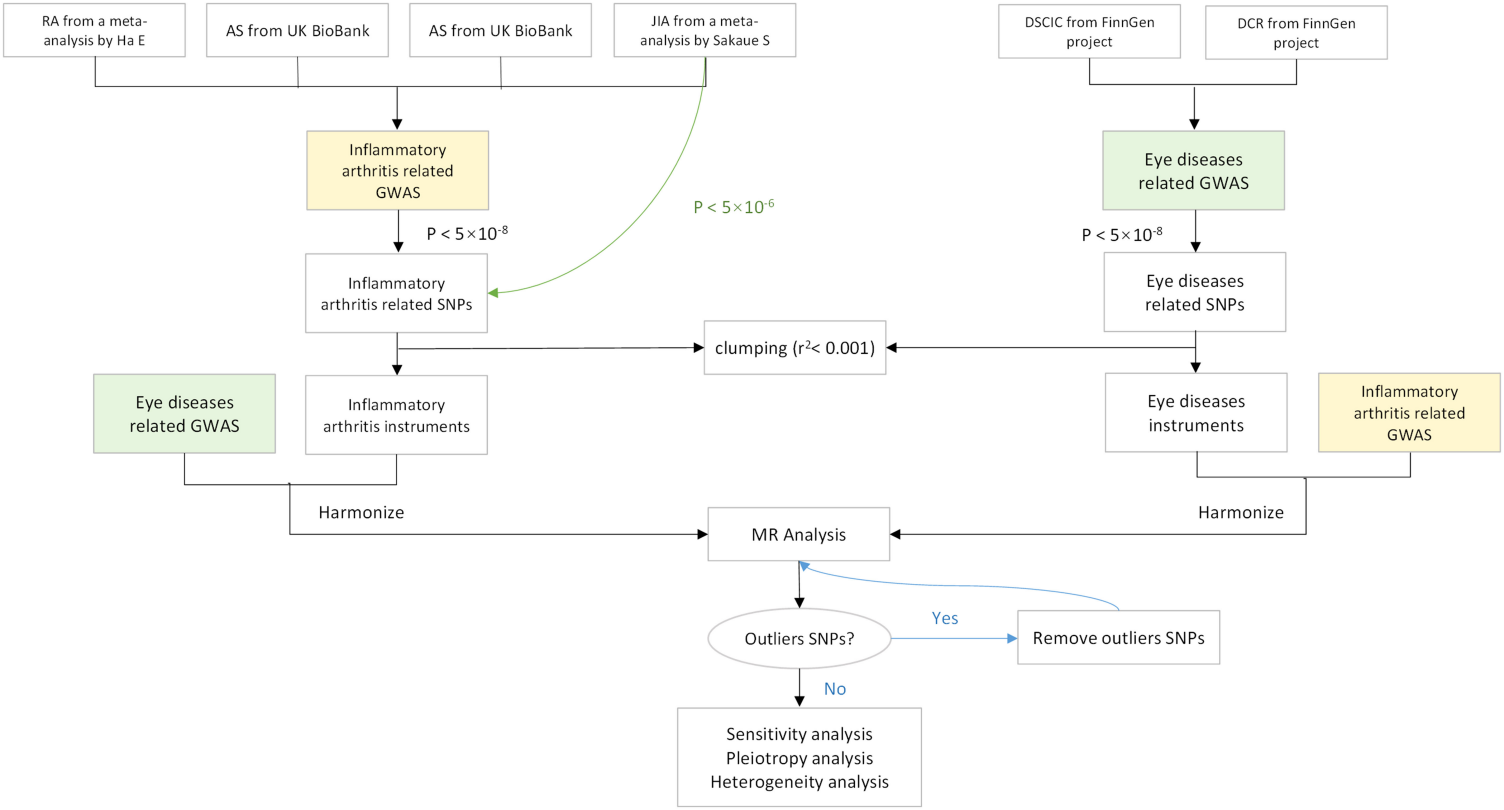
Flowchart of a Mendelian randomization study. Green lines represent separate threshold (*p* < 5×10^-6^) for JIA screening of instrument variables. MR, Mendelian randomization; OR, odds ratio; CI, confidence interval; RA, rheumatoid arthritis; AS, ankylosing spondylitis; PsA, psoriatic arthritis; JIA, juvenile idiopathic arthritis; DSCIC, Disorders of sclera, cornea, iris, and ciliary body; DCR, Disorders of choroid and retina.

We utilized openly accessible data from published studies or GWAS summaries to inform our research. As the study did not utilize primary data, there was no need for ethical approval to be obtained. All the studies that were incorporated have been approved by their respective academic ethics committees and each participant has duly signed the informed consent form. **Figure 1** shows the flow of this study.

### Data sources

A meta-analysis was conducted on the summary statistics of RA association in three extensive case–control collections that comprised a total of 311,292 individuals from Korean, Japanese, and European populations (23). We selected the European populations in the data, including 14,361 cases and 43,923 controls. The GWAS data correlated with AS and PsA were obtained from a meta-analysis (24) from the UK Biobank (https://www.ukbiobank.ac.uk/); all participants were from the European population. The GWAS of AS included 371,733 individuals; meanwhile, PsA data included 407,865 individuals of European ancestry. The summary-level data correlated with JIA included 409,217 individuals of European ancestry. Comprehensive participant descriptions and details are available in the article authored by Sakaue (25).

We found two sets of GWAS data from the FinnGen project released in May 2023 (26), consisting of merely European individuals, which are DSCIC (19,463 cases and 357,814 controls) and DCR (32,708 cases and 344,569 controls). The entirety of SNPs as well as their respective summary data were sourced exclusively from studies that only involved subjects of European ancestry to avoid population stratification bias. Detailed data information is summarized in **Supplementary Table 1**.

### Selection of genetic instruments

To remove SNPs associated with the results, the *p*-value threshold was set to 5 × 10^−8^. Exceptionally, we set the JIA-associated SNP threshold to 5 × 10^−6^, due to the smaller number of SNPs (27, 28). To validate the presence of any SNPs in linkage disequilibrium and assess their independence, a corresponding linkage disequilibrium analysis was conducted. This involved pruning SNPs within a 10,000-kb window with an *r*^2^ < 0.001 threshold. Finally, *F*-statistic was calculated to assess the strength of the selected SNPs according to the following equation:


$$F=\frac{{\text{R}}^{2}(N-1-K)}{(1-{\text{R}}^{2})K}$$

where *R*^2^ is the proportion of exposure variance explained by the instrument variables (IVs), *N* is the number of samples, and *K* is the amount of IVs. A value of the *F*-statistic greater than or equal to 10 indicates non-existence of weak instrument bias (29).

### Mendelian randomization estimates

**Figure 2:**
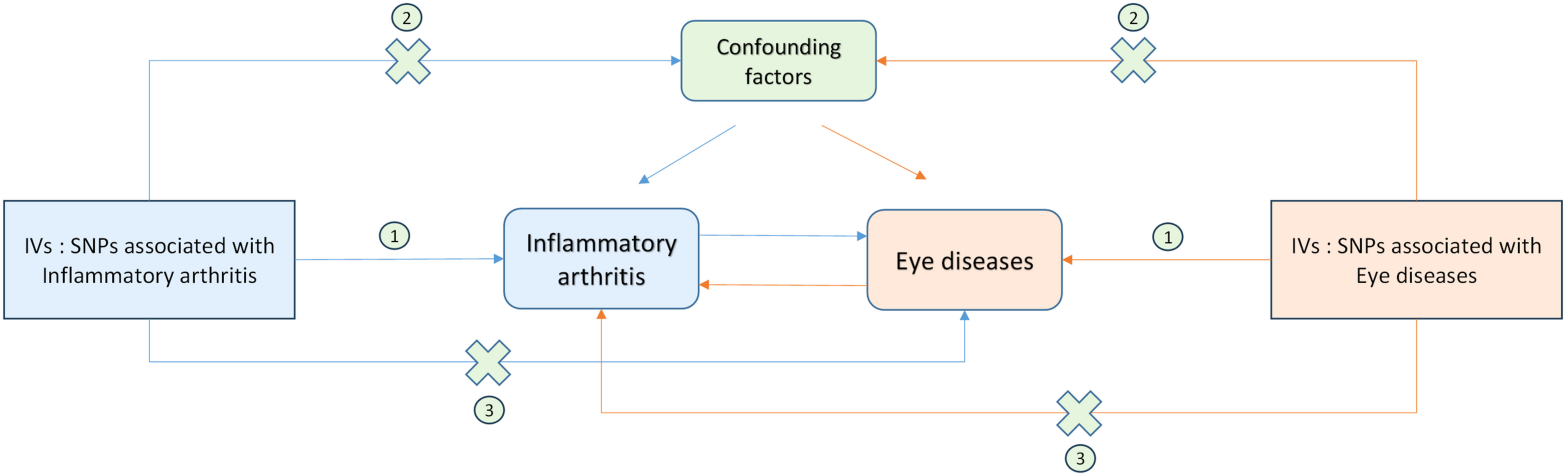
Schematic representation of Mendelian randomization analysis. Inflammatory arthritis and eye diseases as exposure and outcome, respectively, and the instrumental variables must meet three major assumptions. IVs, instrument variables; SNPs, single-nucleotide polymorphisms;**①,** Correlation assumption; **②,** Exclusivity assumption; **③,** Independence assumption.

To examine the genetic causal effect between inflammatory arthritis and eye diseases, we conducted 16 separate two-sample MR analyses, evaluating the association results. The three main assumptions for two-sample MR analysis (22, 30) are as follows (**Figure 2**):

Correlation assumption: Genetic variants should be strongly associated with exposure;

Exclusivity assumption: The variations must be unaltered by any confounding factors that may be associated with either the exposure or the outcome;

Independence assumption: The variants should solely be influenced by the exposure (31).

Since not all genetic variants are considered valid instrumental variables, three robust methods have been proposed. The methods include inverse-variance weighting (IVW), weighted median, and MR-Egger, which were based on various hypotheses for MR analysis. The IVW method was used as the primary method of an MR assessment (32), while weighted median and MR-Egger were employed to augment the precision of IVW estimates.

We conducted an MR-Egger intercept analysis to evaluate potential pleiotropic effects of the selected SNPs utilized as instrumental variables. In the MR-Egger intercept test, the intercept term plays a critical role in assessing whether horizontal pleiotropy exerts any influence on the analysis (33). To reduce heterogeneity in analysis, the assessment excludes SNPs that result in a disproportionate level of heterogeneity compared to what is expected by the MR-PRESSO analysis. A repeated IVW analysis after removing these outlier instruments would be performed, once the outlier instruments were identified. Furthermore, we conducted a “leave-one-out” sensitivity analysis to identify potentially influential SNPs. In this approach, each SNP was systematically excluded one by one from the MR analysis.

R version 4.3.0 with the “TwoSample MR” packages was used for all statistical analyses (34, 35). The Bonferroni-corrected significance threshold was defined as *p*-value < 3.125 × 10^−3^ (correcting 16 outcomes), and *p*-value < 0.05 was regarded as nominally significant.

## Results

### Selection of instrumental variables

The LD-independent SNPs data related to exposures were incorporated in **Supplementary Tables S2**-**S7**. The specified SNPs will be eliminated under the following circumstances: Firstly, SNPs linked to both outcomes and confounding factors will be excluded. Secondly, SNPs that were not directly identified in the outcome GWAS, and for which a suitable proxy in linkage disequilibrium could not be identified during the process of SNP selection, were also excluded. Thirdly, SNPs with ambiguous or palindromic sequences that rendered the correction of non-concordant alleles impractical were also omitted from the analysis. The *F*-statistics of IVs were all greater than 10, implying little evidence of weak instrument bias.

### Estimates of the causal effect

**Figure 3:**
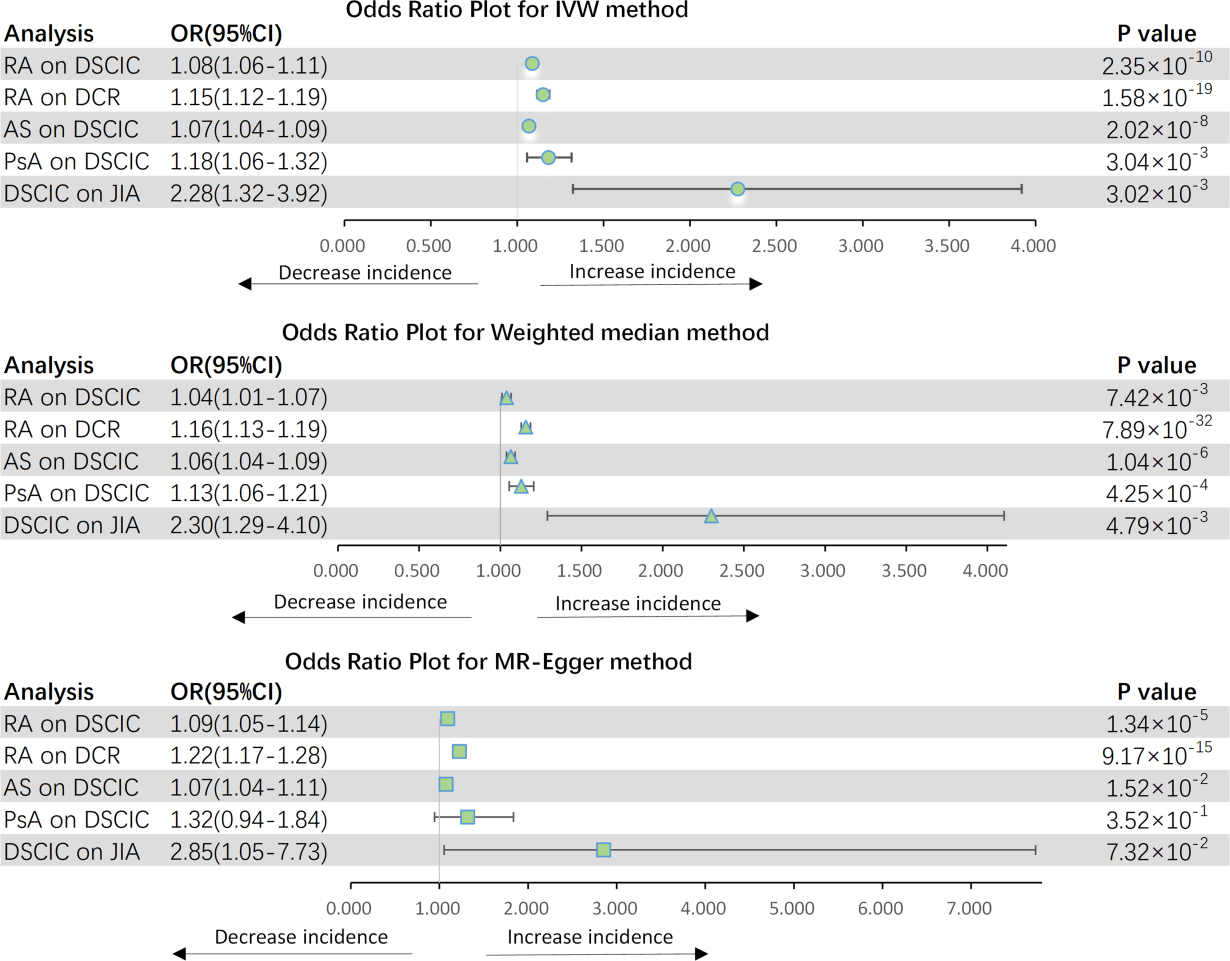
Forest plot for MR analyses. OR, odds ratio; CI, confidence interval; IVW, Inverse-variance weighting; RA, rheumatoid arthritis; AS, ankylosing spondylitis; PsA, psoriatic arthritis; JIA, juvenile idiopathic arthritis; DSCIC, Disorders of sclera, cornea, iris and ciliary body; DCR, Disorders of choroid and retina.

**Figure 3** shows the results of estimating the causal effect of RA on eye diseases. The analysis revealed a statistically significant association between RA and an elevated risk of DSCIC (OR = 1.084, 95% CI: 1.057–1.112, *p* = 2.353 × 10^−10^) as well as DCR (OR = 1.151, 95% CI: 1.116–1.186, *p* = 1.584 × 10^−19^). This association remained consistent across both the MR-Egger and weighted median methods. While Cochran *Q*-derived *p*-values indicated the presence of heterogeneity (*p* > 0.05), the heterogeneity was deemed acceptable given the utilization of the random-effects IVW as the primary method (36). MR-Egger intercept derived *p* > 0.05, indicating that no pleiotropy was detected. After MR-PRESSSO and the leave-one-out plot was detected, no outliers were observed. Then, we further explored AS and PsA association with eye diseases separately. We observed a significant causal effect of AS on DSCIC, with a 6.8% increased risk (OR = 1.068, 95% CI: 1.044–1.094 *p* < 2.024 × 10^−8^). PsA was also found to be causally associated with a 17.9% increased risk of DSCIC (OR = 1.179, 95% CI: 1.057–1.314, *p* = 0.003). However, neither AS (*p* = 0.661) nor PsA (*p* = 0.646) demonstrated a causal effect on DCR. Moreover, there is no observed causal effect of JIA on eye diseases, even when we adjusted the threshold for JIA-associated SNPs to 5 × 10^−6^. This means that our conclusions are not affected.

In addition, just as we expected, DSCIC was associated with the increased risk of JIA (OR = 2.276, 95% CI: 1.322–3.919, *p* = 0.003). After Bonferroni correction, AS and RA were potentially influenced by DSCIC (*p* = 0.031) and DCR (*p* = 0.018), respectively. There are no other eye disease causation affecting inflammatory arthritis.

For both significant and nominal significant estimates, the results of the test of heterogeneity are presented in **Table 1**. In addition, the MR-Egger intercept did not support the existence of horizontal pleiotropy in the analyses of inflammatory arthritis and eye diseases according to **Table 1**. In the MR-PRESSO outlier test, both raw and outlier corrected estimates from MR-PRESSO were consistent with results from the IVW method, demonstrating the stability of the results. Leave-one-out analyses and funnel plots are shown in **Supplementary Figures S1**-**S3**.

**Table 1:** Significant and nominal significant Mendelian randomization estimates for MR analysis between inflammatory arthritis and eye diseases.

	Exposure	Outcome	IVW-derived *p*-value	OR (95% confidence intervals)	Cochran’s *Q*-derived *p*-value	MR-Egger intercept-derived *p*-value
**Significant estimates**	RA	DSCIC	**2.35 × 10^−10^**	1.08 (1.06–1.11)	3.68 × 10^−15^	0.587
	RA	DCR	**1.58 × 10^−19^**	1.15 (1.12–1.19)	2.21 × 10^−6^	0.623
	AS	DSCIC	**2.02 × 10^−8^**	1.07 (1.04–1.09)	6.83 × 10^−4^	0.767
	PsA	DSCIC	**3.04 × 10^−3^**	1.17 (1.06–1.31)	2.70 × 10^−6^	0.609
	DSCIC	JIA	**3.02 × 10^−3^**	2.27 (1.32–3.92)	2.06 × 10^−6^	0.604
**Nominal significant estimates**	DCR	RA	**1.83 × 10^−2^**	2.06 (1.13–3.79)	9.48 × 10^−23^	0.554
	DSCIC	AS	**3.09 × 10^−2^**	33.57 (1.38–816.28)	5.55 × 10^−30^	0.273

IVW-derived p-value < 0.05 is set as nominal significant, whereas < 3.125 × 10^−3^ is set as significant.

MR, Mendelian randomization; Inverse-variance weighted; OR, odds ratio; RA, rheumatoid arthritis; AS, ankylosing spondylitis; PsA, psoriatic arthritis; JIA, juvenile idiopathic arthritis; DSCIC, disorders of sclera, cornea, iris and ciliary body; DCR, disorders of choroid and retina.

IVW is considered to be the most meaningful method for MR analysis. After Bonferroni-corrected, ivw-pvalue < 3.125 × 10−3 is considered to be statistically significant, so we bolded ivw-pvalue in the table to emphasize.

## Discussion

Summary statistics from GWASs were used in this study to explore the causal association between inflammatory arthritis and eye diseases. The eye diseases explored in this study include DSCIC and DCR. We found significant causal associations between RA and the two diseases mentioned above. For AS, we found that it could causally increase the risk of DSCIC but has little causal effect on DCR. In addition, genetically predicted PsA was causally associated with the increased risk of DSCIC. Notably, DSCIC greatly increases the risk of JIA.

Our results indicated that there was a causal relationship between RA and eye diseases, which was consistent with the findings of several published studies (37–40). While various factors could potentially contribute to this association, the patient’s immunologic status appears to be a key determinant. Firstly, there is lymphocytic infiltration. The quantity of Langerhans cells, responsible for presenting antigens, in the central and peripheral cornea of individuals with RA but without ocular manifestations was greater than in those without RA. This result indicates a heightened state of the innate immune system even without apparent eye disorders (41). In addition, lymphocyte infiltration of the lacrimal gland was also discovered in RA patients. Secondly, inflammatory mediators are involved. Inflammatory mediators such as tumor necrosis factor (TNF) are raised in the tears (42) and joints (43) in patients with RA. The significance of TNF in the context of sight-threatening ocular surface diseases in RA patients, such as peripheral ulcerative keratitis and scleritis, was demonstrated through the clinical improvement observed after administering infliximab treatment (44). Furthermore, some soluble immune elements that play a role in RA may be regulated differently at the ocular surface. Individuals who suffer from autoimmune keratitis linked to RA had higher levels of interleukin-17 (IL-17) in their tears (45). However, there was no significant increase in serum IL-17 concentrations in individuals with RA-associated dry eye when compared to those with chronic graft-versus-host disease (46). The results indicate that inflammation of the ocular surface is a result of both a rise in circulatory cytokines due to systemic RA and local immune responses. In fact, inflammatory mediators that cause tissue injury on the ocular surface (including Fas, IL2, IL-6, IL-8, and matrix metalloproteinase) also impair the joint (47, 48). Several crucial steps of immune signaling co-exist in ocular and systemic RA.

For AS and PsA, we found that they could causally increase the risk of DSCIC, which is consistent with the findings of several previous studies (18, 49). The connection between arthritis and uveitis, first described by Brewerton in 1973, was notably associated with the HLA-B27 haplotype (50, 51). Unfortunately, the exact mechanism remains unclear to date. Recent studies have found that polymorphism in the LMP2 gene and HLA-DR8 may play a role in the association between the two diseases, but this still needs to be further explored (52, 53). In addition, common genetic predisposition could also be one of the mechanisms (54).

Notably, DSCIC greatly increases the risk of JIA, which was consistent with the clinical observation of uveitis with JIA concomitant (14, 55, 56). Siiskone found in a cohort study that 99% of JIA-associated uveitis was concentrated in the anterior (14). JIA and childhood uveitis are very difficult to diagnose and may have serious clinical consequences. The incidence of JIA and childhood uveitis is increasing every year (57), and the mechanism of the concomitant pathogenesis of both is not known. Fortunately, the use of bDMARDs has greatly improved the clinical prognosis of pediatric patients with uveitis, and the safety and efficacy of adalimumab have been demonstrated (58). Our study may provide clues to the mechanism and again alert the clinic to the early warning of asymptomatic concomitant disease.

Since many studies were cross-sectional or retrospective, determining the temporal relationship between the onset of inflammatory arthritis and ocular pathology presented a challenge. Despite the emergence of subsequent cohort studies, the influence of confounding variables has yet to be effectively addressed. Consequently, we employed Mendelian randomization analysis to circumvent these limitations, offering a more robust approach to investigate this complex relationship. By incorporating multiple statistical methods, we have bolstered the robustness and reliability of our findings. Furthermore, the GWAS data summary utilized in our study exclusively pertained to individuals of European ancestry, thereby minimizing the possibility of any inherent bias. Most importantly, our findings facilitate scientific research and clinical management of the relationship between inflammatory arthritis and eye diseases.

There are some limitations to our MR analyses. Firstly, since each of the methods we applied in the analyses has its own advantages and disadvantages, there is a potential for inconsistent outcomes. Secondly, the potential horizontal pleiotropy cannot be controlled. Thirdly, the study population only included individuals of European ancestry. Further research is necessary to determine the generalizability of these findings to diverse ethnic groups.

## Conclusion

Our study provided genetic evidence for the causal associations of RA, AS, and PsA with an increased risk of DSCIC. In addition, a causal association between RA and DCR was also identified. Finally, DSCIC greatly increased the risk of JIA.

## Data availability statement

The original contributions presented in the study are included in the article/**Supplementary Material**. Further inquiries can be directed to the corresponding author.

## Ethics statement

Ethical approval was not required for the study involving humans in accordance with the local legislation and institutional requirements. Written informed consent to participate in this study was not required from the participants or the participants’ legal guardians/next of kin in accordance with the national legislation and the institutional requirements.

## Author contributions

YS and XN designed the study, conducted data analyses, and drafted the manuscript. ZL conducted data analyses. DX contributed to the writing. All authors contributed to the article and approved the submitted version.
