# Supplementary material for: Inflammatory arthritis and eye diseases: a Mendelian randomization study
Source: Front Endocrinol (Lausanne). 2023 Oct 9;14:1251167. doi: 10.3389/fendo.2023.1251167 (PMC10591326; doi:10.3389/fendo.2023.1251167)
Supplement: Supplementary file 1 [file DataSheet_1.docx]

Supplementary Information

**Inflammatory arthritis and eye diseases: a Mendelian randomization study**

**Xinlin Nie, MD^1^, Zhaoliang Liu, MD^1^, Dongheng Xie, MD^1^, Yang Sun, MD^1^**

**Figure S1** Scatter plots of significant and nominal significant estimates from genetically predicted (a) rheumatoid arthritis on Disorders of sclera, cornea, iris and ciliary body; (b) rheumatoid arthritis on Disorders of choroid and retina; (c) ankylosing spondylitis on Disorders of sclera, cornea, iris and ciliary body; (d) psoriatic arthritis on Disorders of sclera, cornea, iris and ciliary body; (e) Disorders of choroid and retina on rheumatoid arthritis; (f) Disorders of sclera, cornea, iris and ciliary body on ankylosing spondylitis (g) Disorders of sclera, cornea, iris and ciliary body on juvenile idiopathic arthritis

**Figure S2** Leave-one-out plots of significant and nominal significant estimates from genetically predicted (a) rheumatoid arthritis on Disorders of sclera, cornea, iris and ciliary body; (b) rheumatoid arthritis on Disorders of choroid and retina; (c) ankylosing spondylitis on Disorders of sclera, cornea, iris and ciliary body; (d) psoriatic arthritis on Disorders of sclera, cornea, iris and ciliary body; (e) Disorders of choroid and retina on rheumatoid arthritis; (f) Disorders of sclera, cornea, iris and ciliary body on ankylosing spondylitis (g) Disorders of sclera, cornea, iris and ciliary body on juvenile idiopathic arthritis

**Figure S3** Funnel plots of significant and nominal significant estimates from genetically predicted (a) rheumatoid arthritis on Disorders of sclera, cornea, iris and ciliary body; (b) rheumatoid arthritis on Disorders of choroid and retina; (c) ankylosing spondylitis on Disorders of sclera, cornea, iris and ciliary body; (d) psoriatic arthritis on Disorders of sclera, cornea, iris and ciliary body; (e) Disorders of choroid and retina on rheumatoid arthritis; (f) Disorders of sclera, cornea, iris and ciliary body on ankylosing spondylitis (g) Disorders of sclera, cornea, iris and ciliary body on juvenile idiopathic arthritis
